# Implementation of Industrial Additive Manufacturing: Intelligent Implants and Drug Delivery Systems

**DOI:** 10.3390/jfb9030041

**Published:** 2018-06-29

**Authors:** Jan Sher Akmal, Mika Salmi, Antti Mäkitie, Roy Björkstrand, Jouni Partanen

**Affiliations:** 1 Department of Mechanical Engineering, Aalto University, 02150 Espoo, Finland; mika.salmi@aalto.fi (M.S.); roy.bjorkstrand@aalto.fi (R.B.); jouni.partanen@aalto.fi (J.P.); 2 Department of Otorhinolaryngology–Head and Neck Surgery, University of Helsinki and Helsinki University Hospital, 00029 Helsinki, Finland; antti.makitie@helsinki.fi

**Keywords:** additive manufacturing, 3D printing, biomaterials, parametric modeling, drug delivery systems, embedding, medicine, radiofrequency identification, object memory, internet of things

## Abstract

The purpose of this study is to demonstrate the ability of additive manufacturing, also known as 3D printing, to produce effective drug delivery devices and implants that are both identifiable, as well as traceable. Drug delivery devices can potentially be used for drug release in the direct vicinity of target tissues or the selected medication route in a patient-specific manner as required. The identification and traceability of additively manufactured implants can be administered through radiofrequency identification systems. The focus of this study is to explore how embedded medication and sensors can be added in different additive manufacturing processes. The concept is extended to biomaterials with the help of the literature. As a result of this study, a patient-specific drug delivery device can be custom-designed and additively manufactured in the form of an implant that can identify, trace, and dispense a drug to the vicinity of a selected target tissue as a patient-specific function of time for bodily treatment and restoration.

## 1. Introduction

Additive manufacturing (AM), non-technically known as 3D printing, is a process in which material is added and joined typically on a layer-by-layer basis to make products using digital data of a 3D model, contrary to subtractive manufacturing and formative manufacturing methodologies [1]. Ever since the instigation of the concept in the early 1980s [2], it has been widely used in a broad range of industries, including military, aerospace, automotive, energy, and biomedical fields [3,4]. Tuomi et al. [5] have presented a classification for medical applications of AM that includes medical models for planning, medical aids, tools for medical devices, inert implants, and bio-manufacturing. The focus of this study is to extend the capability of AM in biomedical field via additively-manufacturing implants containing custom-designed drug delivery systems equipped with radiofrequency identification technologies for restoration of bodily harm. To this end, the composition of such a delivery system must be inert and compatible with the human body, which inevitably limits the use to biomaterials. Tappa and Jammalamadaka have classified biomaterials used in AM into four categories that include metal and its alloys, ceramics and carbon compounds, polymers, and composites [6]. According to the ISO/ASTM 52900:2015(en) standard, AM contains seven process categories that mainly differentiate each other through the techniques that they use for the layer-by-layer fabrication of their compatible materials. Hence, the selection of a suitable biomaterial depends on the application that specifies its physical and mechanical properties followed by its compatibility of being additively manufactured through one of the ISO/ASTM additive manufacturing processes. Table 1 presents the biomaterials that are compatible with each of the AM methods that are utilized in this study to provide proof of concept. The use of additively-manufactured biomaterials must always be preceded with medical considerations.

One of the key factors that influence various treatment approaches of human health is drug delivery to the target tissues. Drug delivery typically involves various approaches, systems, technologies, and formulations for the release of an active pharmaceutical ingredient to the patient through different methods and shapes, as required [26,27]. These include, for example, intravenous, intra-articular, intramuscular, cutaneous, subcutaneous, mucosal (intraoral, intranasal, rectal), and pulmonary drug administration. The effectiveness of different drug delivery systems is measured over the course of time through in vitro and/or in vivo testing that may be associated with immediate or long-term side effects. Delivering the drug locally near the damaged tissue has shown good results in several subjects of treatment [28]. This is relevant to enhance the accuracy of a selective treatment and also to shorten the time before the onset of drug effect. Furthermore, side effects, and even complications in healthy tissues, may be avoided when drug release is located close to the target tissue or organ.

Various conditions of the temporomandibular joint (TMJ) affect roughly one quarter of the population, and possible causes include injury, arthritis, and genetics, i.e., certain connective tissue disorders [29]. The therapeutic options are usually non-surgical and managed in primary health care, whereas complicated cases require care by a specialist and surgery is preserved as the last alternative. Recently, tissue engineering has been investigated as an emerging alternative for replacing diseased, displaced, or degenerated tissues in the TMJ [29]. 

Due to the nature of patient-specific anatomical geometry, there is no one-size-fits-all approach to replace a defective part of the body. Therefore, patient-specific planning is needed. There are different methods to acquire patient anatomy into computer aided design (CAD) that is needed for novel medical planning work. 3D scanning methods, such as magnetic resonance imaging (MRI) and computed tomography (CT), complement such a phenomenon by capturing data from the interior, as well as the exterior, of the patient-specific anatomical geometry [30]. The medical imaging data acquired from these methods are stored according to the international standard of digital imaging and communications in medicine (DICOM) format, which resembles a block of raw data that is generally inspected in a 2D manner by practitioners in the field. Hence, 3D thresholding techniques viz. volume-based methods, contour-based methods, and/or point-cloud-based methods are used to acquire the necessary 3D data of the anatomical models [31]. Salmi [32] has effectively evaluated five different anatomical models, including pelvis, knee, backbone, ankle, and heart, through CT scanning followed by volume-based thresholding, 3D modeling and, eventually, AM. 

Radiofrequency identification (RFID) involves detecting and recognizing physical object(s) wirelessly through radio or electromagnetic communications [33,34]. RFID technologies have been used in a vast range of industries such as manufacturing, supply-chain management, and logistics [35]. Its applications also extend to animal tracking [36,37,38], food [39], and health [40]. RFID systems include a transceiver/reader, a transponder/tag, and two antennas for communication [41]. The reader emits radio waves in the range of electromagnetic spectrum to communicate with the tag that carries a unique identifier. The tag can be either active—equipped with a power source—or passive—not equipped with a power source. Passive tags have been widely implanted into animals through hollow needle injection under the skin of the animal [38]. The range of communication between the reader and the tag depends on the power output and the radiofrequency that is used [33]. The radiofrequency range is defined by the ISO standards as low frequency (LF); <135 kHz, high frequency (HF); 13.553–13.567 MHz, and ultra-high frequency (UHF); 433 MHz to 2.45 GHz [41]. The significant progress in low-power semiconductor and microelectronics technologies equipped with the re-usability of the RFID systems and productivity gains have promoted the use of RFID systems to become more affordable [42]. 

The aim of this study is to demonstrate the ability of embedding conceptual medication and RFID transponders into an additively-manufactured implant using different AM techniques. To this end, a conceptual case scenario, including a drug delivery system and an RFID system, was designed to be fabricated via an additively-manufactured implant in order to identify and deliver a specific drug for the therapeutic treatment of various conditions of the TMJ. The concept is presented through conventional materials used for AM, however, it can be implemented using biomaterials that have been studied throughout the literature presented in Table 1.

## 2. Results

**Figure 1:**
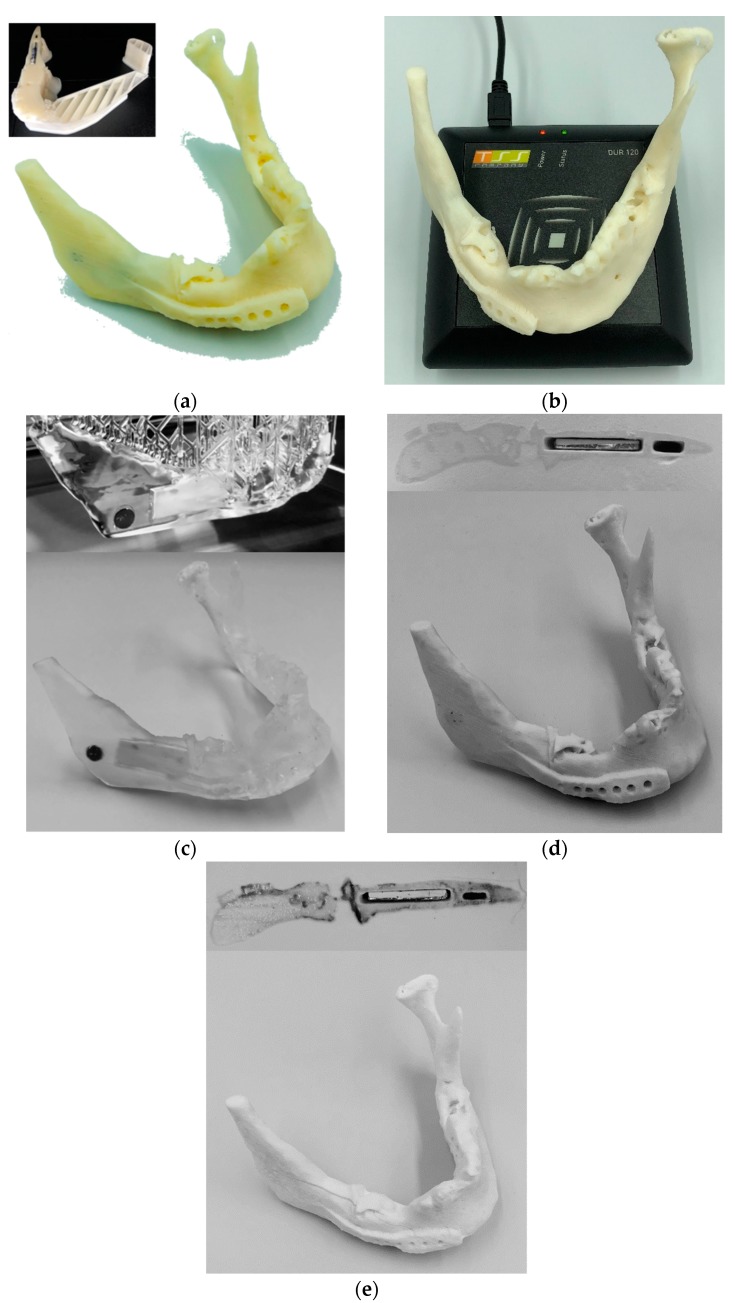
Additively manufactured mandible with embedded conceptual drug and RFID transponders using: (**a**) the material extrusion method; (**b**) RFID transmission with the material extrusion method; (**c**) the vat photopolymerization method; (**d**) the binder jetting method; and (**e**) the powder bed fusion method.

The results of the additively-manufactured implant fused to its corresponding mandible can be seen in Figure 1 using the ISO/ASTM AM methods shown in Table 1. The results of operational communication between the transponder and transceiver can be seen in Table 2. According to these results, all the tested AM methods were able to successfully manufacture the mandible and its implant to a fine detail, including the embedded drug delivery and RFID systems. The level of detail was inversely proportional to the layer thickness of different methods. Upon testing, the intelligent implant was able to identify itself and communicate the necessary patient-specific information in all cases.

## 3. Discussion

The capabilities of additive manufacturing, or 3D printing, have broadened the design freedom and approach of manufacturing throughout the industries. These unique characteristics allow for the implementation of novel, patient-specific applications in the field of medicine. This study allows for custom-designed, additively manufactured drug delivery systems for controlled drug dispensing in direct vicinity of need, which can enhance bodily restoration and lower the risks of unintended side effects of other bodily functions. This is achieved, for example, through embedding the conceptual medication in an implant. In the present case, the right part of a mandible was used as a model, and eight controlled channels were created for dispensing. The drug release or activation will typically involve a variety of mechanisms, i.e., diffusion, degradation, swelling, and affinity-, route-, or dosage-based mechanisms, and needs to be designed separately in each individual application. The dispensing channels can also be manufactured through such biomaterials that dissolve over time and release the drug to the target tissue as required. This study also allows for storage of patient-specific information including the identification of the drug and/or the implant through passive radiofrequency identification transponders, which are also embedded into the implant at the same time as the medication. When the implant has been manufactured with the aforementioned embedded elements, it can be surgically inserted into the required area. Subsequently, the provided concept allows for identifying and reading patient-specific information from outside the body using an RFID transceiver without any invasive procedures. The patient-specific information can represent the digital version of the documentation that patients normally receive postoperatively. This may include information on the patient, implant, and treatment, such as the name of the patient and the embedded medicine, date of birth, date of manufacture of the implant, material of the implant, and/or possible expiration of the implant. This can ensure access to the patient-specific information through the Internet of Things (IoT) even when such physical documentation is lost. The concept also allows for obtaining a digital twin of the implant, for instance, through an identifier, which will enable reproduction of the implant. Contrary to the current industrial practice of a digital twin, this would require a wider range of regulations to be implemented in the field of medicine. 

The parametric nature of drug delivery and RFID cavity systems, developed in this study, allow design automation with a user interface that can aid in 3D modeling even by someone unfamiliar to the art. This ensures a high degree of ease of use for practitioners in the field of medicine who are typically unfamiliar with such design methods. The models allow for controlled drug delivery via the size, length, and number of dispensing channels. To ensure that no material is joined together in the cavity intended for both drug and RFID, a support feature is added to the models that terminates such fusion of material. Since different AM technologies have different parameters, such as minimum layer thickness or resolution, a print clearance feature is also automated in the models. This ensures that there is enough room in the cavity for embedding the intended system.

In this study, the implant, the right part of a mandible, is additively manufactured in connection with its intended anatomical model, the mandible, for demonstration purposes. This can provide a visual aid to the surgeons as a pre-operative planning tool that can serve as a guide for pinpointing the intended incisions and drilling for implantation. Subsequently, the implant with the embedded infrastructure can be printed by itself and implanted through surgery. In this case, the printing times recorded in Table 2 can be up to 80% lower. All the AM methods that were used for this study provided an efficient way of embedding custom drug delivery and RFID systems in implants for controlled delivery and electronic storage of patient-specific information. A few delamination issues were encountered for powder-based AM methods when the machine was paused for too long with an open build chamber. In the case of the BJ method, this is caused due to the usage of a commercial machine that does not allow for variation in binder dispersion. Similarly for the PBF method, this occurs because of the formation of large thermal gradients.

For future developments, the practice of embedding can be easily made free of human error, delamination problems, and issues related to hygiene through a glove system or an automated robotic system where build chambers stay intact. The presented concept can also be extended to other applications that can benefit from embedding drug delivery and/or RFID systems in the medical field. For instance, it is a common practice for those who suffer from severe arthritis in knees to have them replaced by titanium implants. In this case, this concept can aid in providing a medicinal lubricant, i.e., synovial fluid, to the desired contacts for reducing friction and stress shielding. Since knee implants typically have a life cycle of 10–15 years, depending on the case, an RFID transponder can store such patient-specific information for future follow-ups and eventual replacement. Expanding its scope further, the concept can also be used for lubricating moving components in other industries for reduction of fretting and galling wear found in bearings, for instance. RFID systems can aid in identifying parts that are not easily accessible and/or are subject to wear and tear where the use of other identification methods, such as optical recognition, is difficult. This can also provide an infringement-proof solution since the part would have to be destroyed in order to access the RFID. More importantly, this study has investigated four ISO/ASTM-defined AM methods to provide a proof of concept. The rest of the three methods, directed energy deposition (DED), material jetting (MJ), and sheet lamination (SL), can also be studied for future development. 

## 4. Materials and Methods

**Figure 2:**
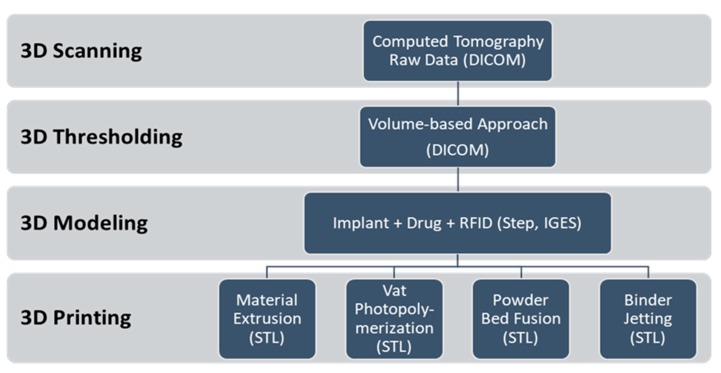
Process of additively manufacturing (3D printing) intelligent implants and drug delivery systems.

For the purpose of this study, four ISO/ASTM-defined AM methods were used to demonstrate the ability of additively manufacturing implants that are equipped with custom-made drug delivery systems and RFIDs. These were material extrusion (ME), vat photopolymerization (VP), binder jetting (BJ), and powder bed fusion (PBF). The process associated with these methods is presented in Figure 2. The process initiates from 3D scanning of the anatomical model using CT scanning in DICOM format. After that, a volume-based 3D thresholding technique was used to segment the voxel data with regard to grayscale algorithm of marching cubes to obtain the patient-specific geometry. Subsequently, the intended implant was 3D modeled with respect to the obtained geometry followed by 3D modeling of incorporated drug delivery and RFID systems. 3D modeling of the latter two systems involves AM parameters that are specific to each method. The following section elaborates on this process according to the methods used in this study.

### 4.1. Mandible Implant

The specimen selected for this study was a mandible that depicts the lower jaw of the human anatomy. The mandible is connected to the TMJ, which is one of the strongest joints in the human body. The data were obtained from a 63-year old patient using GE Lightspeed (GE Healthcare, Chicago, IL, USA) computed tomography with 120 kVp. The scanning was performed in helical mode with layer thickness of 1.25 mm. The total number of layers was 203. The patient had a disorientated mandible with the main part of its right side entirely missing.

**Figure 3:**
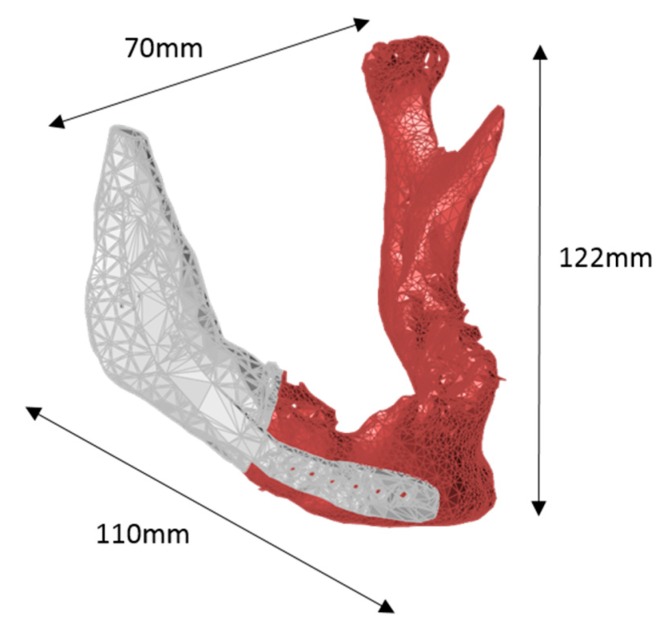
3D scanned, rendered and modeled patient-specific mandible (maroon) connected to its implant (white).

The 3D model of the anatomy was segmented using Osirix (2.7.5, Pixmeo SARL, Geneva, Switzerland) with 500 HU density, 0.5 decimate, and 20 smoothing iterations. First, the mandible was moved to the correct locations. 3D modeling of the implant was done using 3Data Expert (11.0, DeskArtes, Helsinki, Finland) and VisCam RP (3.6, Marcam Engineering, Bremen, Germany) according to the specifications of the surgeon. The starting geometry was mirrored from the left side, which was eventually positioned and formed to fit on the right side. The supporting structure with screw holes was added over the jaw to keep the implant in its designated place. The final model of the mandible connected to its implant can be seen in Figure 3.

### 4.2. Parametric Drug Delivery System

**Figure 4:**
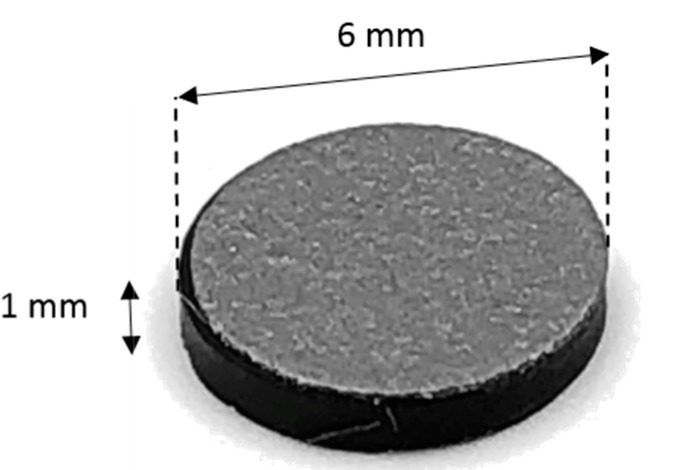
Conceptual tablet drug: HF RFID transponder conferring to ISO18000-3.

**Figure 5:**
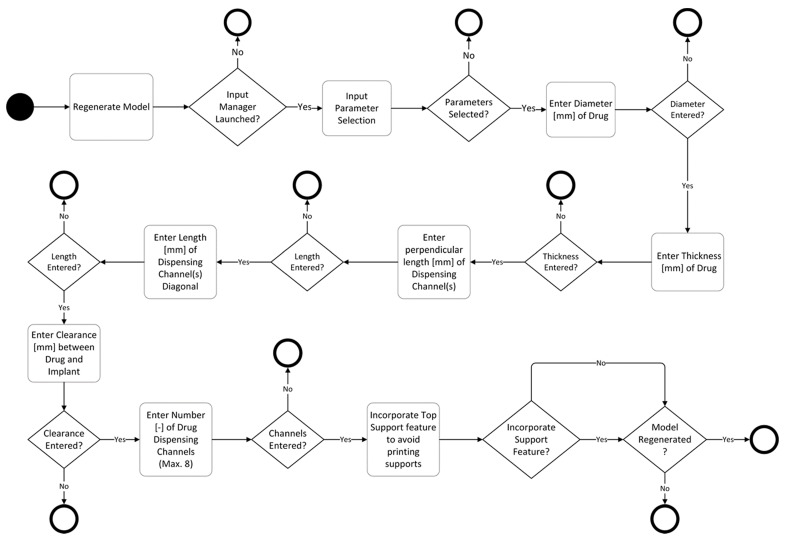
Activity diagram of a parametric drug delivery system consisting of seven design-based input parameters.

**Figure 6:**
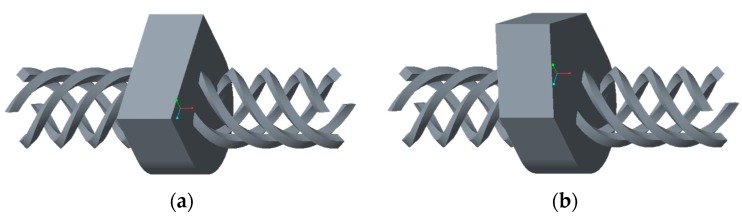
Drug delivery cavity consisting of eight dispensing channels and a support feature that is: (**a**) deactivated for powder-based AM methods; and (**b**) activated for ME and VP methods.

A drug delivery concept was custom-designed using Creo Parametric (3.0, PTC, Needham, MA, USA) through a parametric model that allows for seven custom inputs with regard to the medicine of need. It is based on a conceptual tablet drug, shown in Figure 4, that is, in fact, an HF RFID transponder. The dimensions of the conceptual drug match well with the conventional range of tablets that are taken orally when needed. An activity diagram of this parametric model of the drug cavity can be seen in Figure 5. The model also allows for a controlled rate of drug delivery or dispensing with inputs of number, size, and length of channels that can be seen spiraling away in opposite directions from the drug cavity in Figure 6. Since ME and VP methods require support structures during the manufacturing process to resist gravity for surface overhangs of above 45°, the model is also incorporated with a support feature that removes the automatic support structures of the printer related software, when activated. This feature can be seen in Figure 6a when it is deactivated for powder-based methods of BJ and PBF, and in Figure 6b when it is activated for ME and VP methods. Once the model was recreated in the computer aided design system according to the desired input parameters, it was inserted into the digital implant for Boolean subtraction to form its geometrical cavity using Inspire (2017.3.2, SolidThinking, Troy, MI, USA).

### 4.3. Parametric RFID Systems

**Figure 7:**
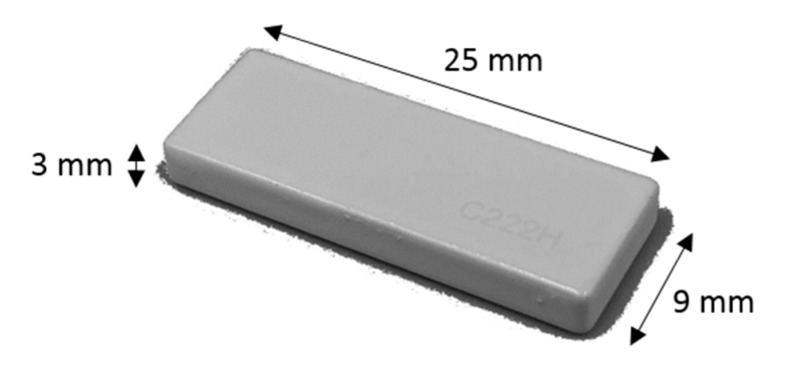
UHF radiofrequency identification transponder conferring to ISO18000-6C.

**Figure 8:**
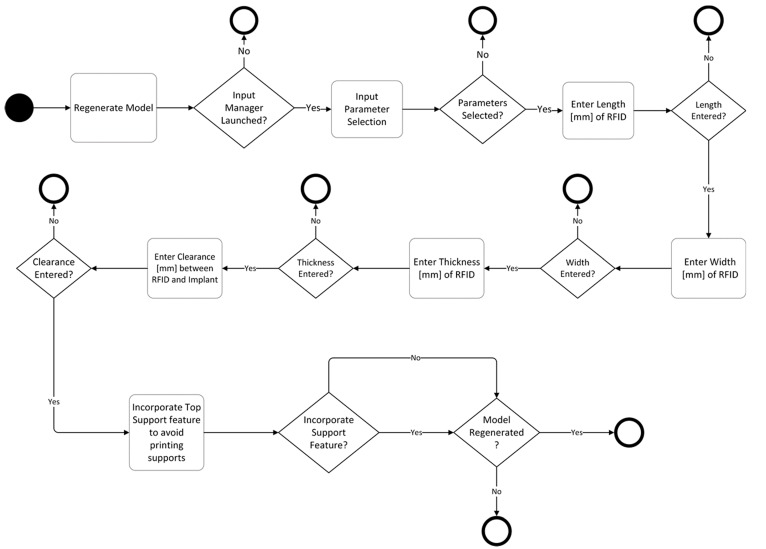
Activity diagram of a parametric RFID system consisting of five design-based input parameters.

**Figure 9:**
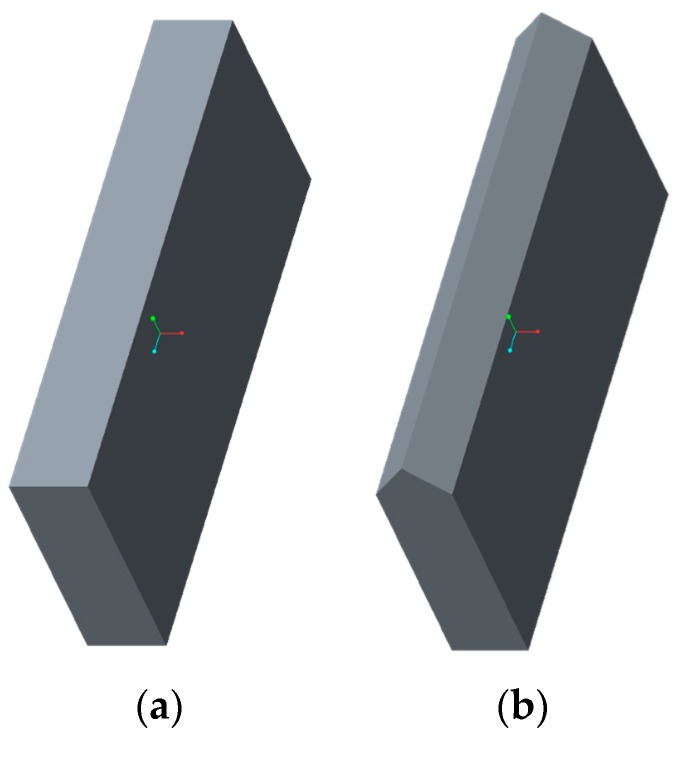
UHF RFID cavity with a support feature (**a**) deactivated for powder-based AM methods and (**b**) activated for ME and VP methods.

For the purpose of this study, HF and UHF RFID systems, presented in Figure 4 and Figure 7, were used for identifying and tracing additively manufactured implants. The operational frequency of the HF transponder was 13.56 MHz while it ranged from 864 to 868 MHz for the UHF transponder. The HF transponder consisted of 1000 bits of user memory that allowed for 125 ASCII characters for patient-specific information. Similarly, the UHF transponder had 512 bits for 64 ASCII characters. The UHF transponder consisted of lower user memory than HF. However, it had a longer communication range. A parametric model of UHF RFID cavity was custom-designed using Creo Parametric (3.0, PTC, Needham, MA, USA) that allows for five input parameters, as shown in the activity diagram in Figure 8. Similarly to the parametric drug delivery model, it also contains a support feature that removes the automatic support structures of the printer-associated software. The status of this feature for different AM methods can be seen in Figure 9. When recreated with the correct input parameters, the top height (*z*-axis) of the model, shown in Figure 9a, was aligned with the top height (*z*-axis) of the drug delivery system, shown in Figure 6a, and inserted into the implant digitally. Subsequently, a Boolean subtraction was performed to form its cavity into the implant.

### 4.4. Additive Manufacturing

The AM process was initiated when 3D modeling of the implant, anatomical model, drug delivery system, RFID system, and their unification was completed. 3D modeled data were eventually converted to AM compatible standard tessellation language (STL) format. Due to the complexity of the voxel data generated from the 3D thresholding operations, the STL file was put through a series of repairing operations involving correction of problematic vertices, faces, and non-manifold edges. The software packages that were used for repair were GrabCAD Print (1.17.14, Stratasys, Eden Prairie, MN, USA), Preform (2.15.1, Formlabs, Somerville, MA, USA), Netfabb (Standard 2018.0, Autodesk, San Rafael, CA, USA), and Meshlab (2016.12, open-source, Pisa, Italy). Table 3 presents the properties and parameters associated with different AM methods that were used for this study. A maximum amount of infill density available for each corresponding AM machine was used. 

The conceptual drug and the RFID transponder were manually embedded into the implant by gaining access to the build chambers when needed. This was achieved by inserting a specific pause into the G-code of the machines at the maximum vertical (*z*-axis) height of the drug and RFID cavities, shown in Figure 6a and Figure 9a, which had been aligned. The leftover loose powder from the drug and RFID cavities was removed using a combination of mini-vacuum, compressed air, and tweezers for powder-based AM methods. No material was removed from the respective cavities of ME and VP methods due to the embedded support feature of the parametric models.

## 5. Conclusions

In this study, different AM techniques were used to additively manufacture intelligent implants containing embedded custom-made drug delivery systems and RFID systems. These techniques included ME, VP, BJ, and PBF. The process associated with these techniques involves 3D scanning, 3D thresholding, 3D modeling and, finally, 3D printing/AM. A patient-specific mandible, i.e., lower jaw, was additively manufactured using the aforementioned techniques by following the process defined in this study. Custom-made drug delivery systems were parametrically modeled allowing for seven input parameters related to the AM machine, geometry, and controlled release of medicine. Similarly, five inputs are required for parametric modeling of RFID systems that allow for identification and digital storage of patient-specific information enabling the creation of a digital twin via IoT. Both parametric models allow ease of use through a guided interface for practitioners in the field of medicine who may or may not be familiar with the art of design. Both systems also contain a support feature that removes the automated support generation of printer-associated software. This allows cavities of both systems to be empty for respective embedding for support structure-based AM methods, such as ME and VP. All four AM processes used in this study provided effective methods of embedding medication, as well as RFID systems, into implants. The study was limited to conventional polymers of the ME method, VP method, and PBF method, and the gypsum-like composite of the BJ method. The concept can be implemented through the use of biomaterials that are examined throughout this study for each AM method presented in Table 1. This paper serves as a proof of concept that AM can be used to manufacture intelligent implants containing embedded medicine and RFID systems for controlled drug release and digital storage of patient-specific information that may increase bodily restoration. The concept can also be extended to other fields of industry for controlled release of materials and their identification through RF.

## Figures and Tables

**Table 1:** Biomaterials with respect to the ISO/ASTM AM methods.

ISO/ASTM Additive Manufacturing Method	Compatible Biomaterials	Source
Material Extrusion (ME)	Polycaprolactone (PCL) Polylactide (PLA) Polycarbonate (PC) Polyvinyl alcohol (PVA) Polylactic-co-glycolic acid (PLGA)	[6,7,8,9]
Binder Jetting (BJ)	Poly-ε-caprolactone Polylactide-coglycolide Poly-l-lactic acid $\begin{array}{c} Starch\\ Dextran\\ Gelatin \end{array}\}+water$ Peptides Proteins (e.g., fibrinogen, collagen) Polysaccharides (e.g., hyaluronan, alginate) DNA plasmids Living cells	[10,11,12,13,14,15]
Vat Photopolymerization (VP)	Polypropylene fumarate (PPF) Polycaprolactone (PCL) Dental SG Resin	[9,16,17]
Powder Bed Fusion (PBF)	PCL Polyether ether ketone (PEEK) PVA + Hydroxyapatite (HA) PEEK + HA Calcium phosphate Titanium TiAL6V4 ELI	[18,19,20,21,22,23,24,25]

**Table 2:** Results of embedded RFID systems.

ISO/ASTM AM Method	Embedded ISO HF RFID Data Communicated [Yes/No]	Embedded ISO UHF RFID Data Communicated [Yes/No]
ME	Yes	Yes
VP	Yes	Yes
BJ	Yes	Yes
PBF	Yes	Yes

**Table 3:** Properties and parameters of different AM methods.

ISO/ASTM AM Method	Machine	Software	Material	Layer Thickness	Printing Time	Other Parameters	Post Processing
ME	Stratasys Uprint SE Plus	GrabCAD Print	ABS+	0.254 mm	8 h 44 min	Model interior fill: solid Model support fill: SMART	Manual and dissolvable support removal
VP	Formlabs Form 2	Preform	Clear Resin V4	0.1 mm	6 h 17 min	Support density: 1.00 Support point size: 0.60 mm	Manual support removal
BJ	3D Systems Zprinter 450	ZPrint	ZP 150	0.1 mm	4 h 22 min	Bleed compensation: on	Compressed air depowdering and drizzle infiltration
PBF	Academic Printer	RepliSLS3D	PP	0.2 mm	2 h 54 min	Laser power: 16.5 W Scan speed: 2250 mm/s Hatch distance: 0.4 mm Energy density: 0.092 J/mm^3^	Compressed air depowdering
